# Knowledge and Awareness of the Relationship Between Heart Failure and Chronic Uncontrolled Hypertension Among Communities in the Aseer Region of the Kingdom of Saudi Arabia

**DOI:** 10.7759/cureus.45859

**Published:** 2023-09-24

**Authors:** Nouf Alhammadi, Abdullah A Alaskari, Abdulrahman A Almaymoni, Abdulsalam A Asiri, Ahmed A Khuzayyim, Ali M Alasiri, Faisal N Almuidh, Khalid A Asiri, Osama A Asiri, Ahmed H Alshammari, Ahmed S Al Zomia

**Affiliations:** 1 Rheumatology, King Khalid University, Abha, SAU; 2 College of Medicine, King Khalid University, Abha, SAU; 3 Internal Medicine, King Khalid University, Abha, SAU; 4 Medicine, King Khalid University, Abha, SAU; 5 Inventory Control, Rafha Maternity and Children Hospital, Rafha, SAU

**Keywords:** saudi arabia, aseer region, health education, health literacy, heart failure, hypertension

## Abstract

Background: The primary objective of this study was to assess the awareness among respondents in Aseer, Saudi Arabia, regarding the link between uncontrolled hypertension (HTN) and the potential development of heart failure (HF). Furthermore, we examined variations in the knowledge of essential information based on whether participants had a history of HF or HTN.

Methods: Employing a snowball sampling method, we conducted a prospective online cross-sectional survey targeting adults aged 18 years and above, encompassing both males and females. The survey participants were residents of the Aseer region with access to the internet.

Results: A total of 418 responses were included in the final analysis; 26.8% were aged 45-55 years, 53.8% were males, 69.1% held a university degree, 17.5% were healthcare workers (HCWs), and 26.8% reported having HTN. There was a statistically significant difference between respondents with and without HF regarding knowledge about uncontrolled HTN and its definition. Television and the internet were the most prominent sources of information, with 31.8% and 35.6%, respectively. Of the responders, 50% knew that uncontrolled HTN can lead to HF. Gender differences were significant, with 51.20% of females and 48.80% of males recognizing this link (p = 0.039). HCWs showed higher awareness compared to non-HCWs (70.81% vs. 29.19%, p < 0.001). HF awareness significantly impacted the respondent’s knowledge (80.38%, p < 0.001). Those knowledgeable about uncontrolled HTN were more likely to be aware of this connection (60.29% vs. 25.84%, p < 0.001).

Conclusions: A large sector of the general population did not know that uncontrolled HTN may cause HF, especially those free from both conditions.

## Introduction

Hypertension (HTN) is a preventable and generally manageable condition; however, the absence of treatment can lead to serious and life-threatening complications such as heart, kidney, and brain disorders [1]. HTN is the most common health problem in developed and developing countries [1]. It is a critical concern in developing countries where the health landscape is shifting from communicable to non-communicable diseases [2]. HTN is the leading risk factor for cardiovascular disease (CVD), the leading cause of mortality worldwide, and continues to represent a significant global health burden [3]. The burden of HTN and its associated complications is progressively linked with age [4].

According to the Eighth Joint National Committee criteria (2014), uncontrolled HTN was classified as having a systolic blood pressure (SBP) greater than or equal to 140 mmHg and a diastolic blood pressure (DBP) greater than or equal to 90 mmHg [5]. Uncontrolled HTN was linked to a heightened risk of developing incident heart failure (HF), particularly significant for individuals with an SBP ≥ 160 mmHg, except for those with chronic kidney disease (CKD), where the risk appeared to rise at an SBP ≥ 140 mmHg. Moreover, uncontrolled HTN was also connected with an elevated risk of various other cardiovascular morbidities and mortality [6].

Alenazi and Alqahtani [7] analysed data from a 2017 household health survey in Saudi Arabia, revealing a 9.2% prevalence of HTN among 15-year-olds, higher in women (10.0%) than men (8.5%). The prevalence increased with age, with 55.3% in women and 48.0% in men. The prevalence of HTN varied significantly between regions. Between 1995 and 2000, another study was conducted in Saudi Arabia on people aged 30-70 years; the prevalence of HTN was 26.1% among men and 23.9% among women [8]. A review of 14 studies on HTN and dyslipidaemia in Saudi Arabia identified data gaps in awareness, screening, diagnosis, treatment, adherence, and control. The HTN prevalence was 14.0-41.8%, while the dyslipidaemia prevalence was 12.5-62.0%. The HTN screening rate was 100%, but only 27.6-61% were aware, underwent diagnosis, received treatment, and achieved blood pressure (BP) control [9].

According to research, patients with HTN frequently do not have a thorough awareness of their disease despite having a reasonably broad understanding of it. Critical elements like the importance of SBP regulation and routine BP monitoring sometimes go unnoticed [10]. As a result, HTN patients need teaching and intervention programmes. According to a study, insufficient perceptions of good health and few doctor visits, particularly among black men, are significant barriers to knowledge, treatment, and control [11].

According to the study's hypothesis, the people in Saudi Arabia's Aseer region lacked sufficient understanding and awareness of the complex relationship between chronic uncontrolled HTN and HF. The primary objective of this study was to assess the awareness among respondents in Aseer, Saudi Arabia, regarding the link between uncontrolled HTN and the potential development of HF. Furthermore, we examined variations in the knowledge of essential information based on whether participants had a history of HF or HTN.

## Materials and methods

Study setting

Aseer is one of Saudi Arabia's administrative regions in the country's southwestern corner, with Abha serving as the emirate's seat. The research was carried out in the Aseer area of Saudi Arabia, which encompassed 16 governorates, including Abha, Muhayil, An-Namas, Billasmar, Billahmar, Balqarn, Bareq, Bishah, Khamis Mushayt, Rijal Alma, Tathlith, Sarat Ubaidah, Ahad Rifaydah, Al-Majarah, and Al-Harajah.

Study design and participants

Employing a snowball sampling method, we conducted a prospective online cross-sectional survey targeting adults aged 18 years and above, encompassing both males and females. The survey participants were residents of the Aseer region with access to the internet. A minimal sample size of 384 was calculated using Epi Info (Centers for Disease Control and Prevention, Atlanta, Georgia) to meet a 95% confidence level with a margin of error of 0.05. This computation was based on a population proportion of 0.50 for those with extensive knowledge of HTN and its association with HF. To accommodate for a 10% nonresponse rate, the final sample size was reduced to 420 individuals.

Study outcome

The study’s main goal was to determine how well responders knew that HF could result from uncontrolled HTN. Additionally, we contrasted respondents' levels of fundamental information according to whether they had HF or HTN.

Data collection

The questionnaire collected information on age, sex, education, status of healthcare workers (HCWs), prevalence of HTN, use of antihypertensive drugs, and the presence of HF. The second section of the questionnaire investigated respondents' understanding of HTN, its symptoms, the symptoms of HF, the connection between uncontrolled HTN and HF, the impact of controlling BP on preventing HF, and lifestyle modifications for regulating high BP. Symptoms of HTN (blurring vision, chest pain, headache, and dizziness), HF symptoms (dyspnoea, arrhythmia, lower limb oedema, and fatigue), and lifestyle modifications for controlling high BP were investigated. This section asked participants about lifestyle changes that can help control high BP. The options included reducing anxiety, complying with prescribed medications, increasing physical activity, and having a low-sodium diet.

Ethical considerations

The research goals were thoroughly explained to the study participants before participation, and each participant gave their written consent. Ethical approval was obtained from the Research Ethics Committee of King Khalid University to ensure the respect of moral guidelines (IRB: ECM#2023-2306). The principles specified in the Declaration of Helsinki were strictly adhered to throughout the research process.

## Results

We collected 473 responses. Of them, 55 responses were excluded as they did not fulfil the inclusion criteria. A total of 418 responses were included in the final analysis. Regarding age distribution, 15.6% were 18-25 years old, 10.3% were aged 26-35 years, 22.7% fell between 36 and 45 years, and the largest segment was between 45-55 years, accounting for 26.8%. Regarding gender, 46.2% identified as female, while 53.8% identified as male. In terms of education, a small proportion were illiterate (0.2%), 0.5% had primary education, 3.6% had preparatory education, 26.6% had secondary education, and nearly two-thirds (69.1%) held a university degree. Concerning HCWs, 82.5% responded negatively, while 17.5% confirmed their status as HCWs. Regarding HTN, 73.2% did not have HTN, whereas 26.8% reported having HTN. Among those with HTN, 73.2% were not on antihypertensive drugs, 10.3% had HTN but did not receive medication, and 16.5% were taking antihypertensive drugs (Table 1).

**Table 1:** Sociodemographic characteristics of the studied population

Variables (n = 418)		Frequency	Per cent
Age	18–25 years	65	15.6
	26–35 years	43	10.3
	36–45 years	95	22.7
	45–55 years	112	26.8
Sex	Female	193	46.2
	Male	225	53.8
Education	Illiterate	1	0.2
	Primary	2	0.5
	Preparatory	15	3.6
	Secondary	111	26.6
	University	289	69.1
Are you a healthcare worker	No	345	82.5
	Yes	73	17.5
	Total	418	100
Have hypertension	No	306	73.2
	Yes	112	26.8
Have antihypertensive drugs	Do not have antihypertensive drugs	306	73.2
	Do not receive antihypertensive drugs	43	10.3
	Yes, on antihypertensive drugs	69	16.5
Have heart failure	No	366	87.6
	Yes	52	12.4

In terms of knowing about uncontrolled HTN, a higher percentage of participants with HTN (31.70%) were aware of it compared to those without HTN (23.10%), showing a trend toward significance (p = 0.05). Similarly, when asked about the definition of uncontrolled HTN, participants in both groups primarily associated it with continuously elevated BP. The awareness of HF was more prevalent among those without HTN (74.40%) compared to those with HTN (25.60%) (p = 0.44). When asked about the meaning of HF, the majority understood it as the heart becoming weak and unable to pump blood, while some associated it with heart arrest or a fast heartbeat. In particular, a higher percentage of participants without HTN (77.90%) expressed uncertainty compared to those with HTN (22.10%).

On the other hand, there was a statistically significant difference between those who knew about uncontrolled HTN for patients with HF and those without HF (16.7% vs. 9.2%, p = 0.023). A high proportion of respondents without HF knew the correct definition of uncontrolled HTN compared to respondents without HTN (87.2% vs. 12.8%); however, this difference was not significant. Statistically significant differences were observed for the response "yes" to the question, "Do you know about HF?" (25.7% vs. 5.2%, p < 0.001). A similar difference was observed with respect to the correct definition of HF (84.90 vs. 15.1%, p = 0.014) (Table 2).

**Table 2:** Exploring awareness and understanding of HTN and HF among Aseer residents HTN: hypertension; HF: heart failure.

Studied variables			HTN			HF		
			No	Yes	p	No	Yes	p
Know about uncontrolled hypertension	No	n	183	55	0.05	216	22	0.023
		%	76.90	23.10		90.80	9.20	
	Yes	n	123	57		150	30	
		%	68.30	31.70		83.30	16.70	
Definition of uncontrolled hypertension	High blood pressure that cannot be controlled	n	165	62	0.971	198	29	0.894
		%	72.70	27.30		87.20	12.80	
	Blood pressure that can be controlled at a certain level	n	73	26		88	11	
		%	73.70	26.30		88.90	11.10	
	Continuously elevated blood pressure	n	59	20		68	11	
		%	74.70	25.30		86.10	13.90	
	Blood pressure that is continuously low	n	9	4		12	1	
		%	69.20	30.80		92.30	7.70	
Know about heart failure	No	n	201	69	0.44	256	14	<0.001
		%	74.40	25.60		94.80	5.20	
	Yes	n	105	43		110	38	
		%	70.90	29.10		74.30	25.70	
What is meant by heart failure?	The heart becomes weak and cannot pump blood	n	206	73	0.051	237	42	0.014
		%	73.80	26.20		84.90	15.10	
	Heart arrest	n	13	10		20	3	
		%	56.50	43.50		87.00	13.00	
	Heart beats fast	n	6	6		9	3	
		%	50.00	50.00		75.00	25.00	
	Not sure	n	81	23		100	4	
		%	77.90	22.10		96.20	3.80	

**Figure 1:**
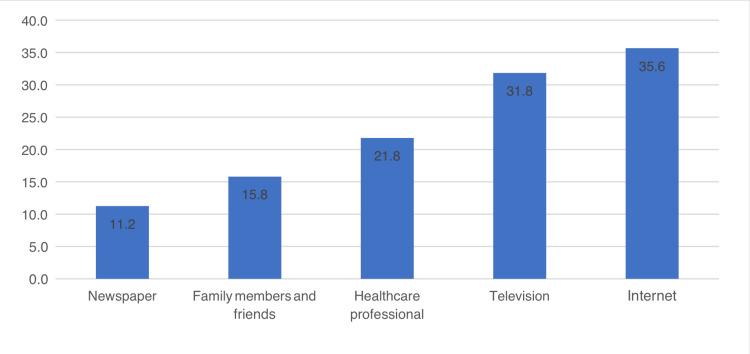
Source of health information

The percentage distribution revealed that television and the internet were the most prominent sources of information, with 31.8% and 35.6%, respectively. Healthcare professionals also played a significant role, accounting for 21.8% of the information. Family members and friends contributed 15.8% of the information; newspapers were the least common source, representing 11.2% (Figure 1).

Gender differences were significant, with 51.20% of women and 48.80% of men recognizing the link (p = 0.039). Education levels did not significantly impact awareness. HCWs showed higher awareness compared to non-HCWs (70.81% vs. 29.19%, p < 0.001). The presence of HTN did not significantly affect awareness. Those knowledgeable about uncontrolled HTN were more likely to be aware of this connection (60.29% vs. 25.84%, p < 0.001). The definition of uncontrolled HTN varied, with no significant awareness correlation. HF awareness significantly impacted respondents' knowledge. Those who understood "heart became weak and cannot pump blood" were more accurate (80.38%, p < 0.001) (Table 3).

**Table 3:** Analysis of HTN and HF awareness: exploring factors influencing knowledge and perception HTN: hypertension; HF: heart failure.

Studied variables		Total		Do not know HTN causes HF		Know HTN causes HF		
		N	%	N	%	N	%	p
Age	18–25	65	15.55	28	13.40	37	17.70	0.517
	26–35	43	10.29	20	9.57	23	11.00	
	36–45	95	22.73	50	23.92	45	21.53	
	45–55	112	26.79	62	29.67	50	23.92	
Sex	Female	103	24.64	107	51.20	86	41.15	0.039
	Male	193	46.17	102	48.80	123	58.85	
Education	Illiterate	1	0.24	0	0.00	1	0.48	0.882
	Primary	2	0.48	1	0.48	1	0.48	
	Preparatory	15	3.59	8	3.83	7	3.35	
	Secondary	111	26.56	57	27.27	54	25.84	
	University	289	69.14	143	68.42	146	69.86	
Healthcare workers	No	345	82.54	197	94.26	148	70.81	<0.001
	Yes	73	17.46	12	5.74	61	29.19	
Have hypertension	No	306	73.21	161	77.03	145	69.38	0.077
	Yes	112	26.79	48	22.97	64	30.62	
Know about uncontrolled hypertension	No	238	56.94	155	74.16	83	39.71	<0.001
	Yes	180	43.06	54	25.84	126	60.29	
Definition of uncontrolled hypertension	High blood pressure that cannot be controlled	227	54.31	110	52.63	117	55.98	0.120
	Blood pressure that can be controlled at a certain level	99	23.68	54	25.84	45	21.53	
	Continuously elevated blood pressure	79	18.90	35	16.75	44	21.05	
	Blood pressure that is continuously low	13	3.11	10	4.78	3	1.44	
Know about heart failure	No	270	64.59	185	88.52	85	40.67	<0.001
	Yes	148	35.41	24	11.48	124	59.33	
What is meant by heart failure	The heart becomes weak and cannot pump blood	279	66.75	111	53.11	168	80.38	<0.001
	Heart arrest	23	5.50	9	4.31	14	6.70	
	Heart beats fast	12	2.87	5	2.39	7	3.35	
	Not sure	104	24.88	84	40.19	20	9.57	

Participants who were unaware of the HTN-HF link had lower awareness of certain symptoms than those who were aware: blurring vision (67.94% vs. 53.59%, p = 0.003), chest pain (79.43% vs. 61.72%, p < 0.001), headache (34.45% vs. 21.05%, p = 0.002), and dizziness (66.5% vs. 47.37%, p < 0.001). Participants who were unaware of the HTN-HF link generally had a lower awareness of HF symptoms: dyspnoea (58.37% vs. 33.49%, p < 0.001), arrhythmia (77.51% vs. 60.29%, p < 0.001), lower limb oedema (70.33% vs. 45.45%, p < 0.001), and fatigue (64.59% vs. 40.19%, p < 0.001). Participants unaware of the HTN-HF link had a significantly worse understanding of the relationship with lifestyle measures. Lifestyle measures for HTN control were generally better understood by the aware group: reduce anxiety (45.45% vs. 28.23%, p < 0.001), compliance with medication (44.02% vs. 65.07%, p < 0.001), increase physical activity (41.63% vs. 57.42%, p = 0.001), and low-sodium diet (51.20% vs. 69.38%, p < 0.001) (Table 4).

**Table 4:** Knowledge and awareness of HTN–HF link: a comprehensive analysis by symptom recognition and lifestyle understanding HTN: hypertension; HF: heart failure.

Variables		Total		Do not know HTN causes HF		Know HTN causes HF		p
		N	%	N	%	N	%	
What are the symptoms of hypertension?								
Blurring vision	No	254	60.77	142	67.94	112	53.59	0.003
	Yes	164	39.23	67	32.06	97	46.41	
Chest pain	No	295	70.57	166	79.43	129	61.72	<0.001
	Yes	123	29.43	43	20.57	80	38.28	
Headache	No	116	27.75	72	34.45	44	21.05	0.002
	Yes	302	72.25	137	65.55	165	78.95	
Dizziness	No	238	56.94	139	66.51	99	47.37	<0.001
	Yes	180	43.06	70	33.49	110	52.63	
What are the symptoms of heart failure?								
Dyspnoea	No	192	45.93	122	58.37	70	33.49	<0.001
	Yes	226	54.07	87	41.63	139	66.51	
Arrhythmia	No	288	68.90	162	77.51	126	60.29	<0.001
	Yes	130	31.10	47	22.49	83	39.71	
Lower limb oedema	No	242	57.89	147	70.33	95	45.45	<0.001
	Yes	176	42.11	62	29.67	114	54.55	
Fatigue	No	219	52.39	135	64.59	84	40.19	<0.001
	Yes	199	47.61	74	35.41	125	59.81	
Which of the following lifestyles control high blood pressure?								
Reduce anxiety	No	264	63.16	150	71.77	114	54.55	<0.001
	Yes	154	36.84	59	28.23	95	45.45	
Compliance with the prescribed medication	No	190	45.45	117	55.98	73	34.93	<0.001
	Yes	228	54.55	92	44.02	136	65.07	
Increase physical activity	No	211	50.48	122	58.37	89	42.58	0.001
	Yes	207	49.52	87	41.63	120	57.42	
Having low-sodium diet	No	166	39.71	102	48.80	64	30.62	<0.001
	Yes	252	60.29	107	51.20	145	69.38	

Participants who were aware that HTN causes HF displayed different perceptions of risk factors compared to those who did not know. Among those who knew, the percentages of participants who considered certain factors as risk factors for HF were the following: diabetes mellitus (71.8%), family history of HF (67.5%), smoking (59.3%), obesity (52.6%), and HTN (41.1%). In contrast, among those who did not know, the percentages were lower for each factor: diabetes mellitus (28.2%), family history of HF (32.5%), smoking (40.7%), obesity (47.4%), and HTN (58.9%) (Table 5).

**Table 5:** Risk factors for HF: a comparative analysis of awareness among different HTN–HF knowledge groups HTN: hypertension; HF: heart failure.

Think the following are risk factors for heart failure		Total		Do not know HTN causes HF		Know HTN causes HF		
		N	%	N	%	N	%	p
Diabetes mellitus	No	256	61.2	150	71.8	106	50.7	<0.001
	Yes	162	38.8	59	28.2	103	49.3	
Family history of heart failure	No	243	58.1	141	67.5	102	48.8	<0.001
	Yes	175	41.9	68	32.5	107	51.2	
Smoking	No	195	46.7	124	59.3	71	34.0	<0.001
	Yes	223	53.3	85	40.7	138	66.0	
Obesity	No	184	44	110	52.6	74	35.4	<0.001
	Yes	234	56	99	47.4	135	64.6	
Hypertension	No	124	29.7	86	41.1	38	18.2	<0.001
	Yes	294	70.3	123	58.9	171	81.8	

## Discussion

Uncontrolled HTN is a serious public health concern in both developed and developing countries. BP control rates remain alarmingly low (less than 30% meet the target of 140/90 mmHg) despite improvements in diagnostic methods and treatment options for HTN, which have shown demonstrable benefits in reducing cardiovascular-related health risks. This is seen even in those who have been diagnosed with HTN and are taking medication to lower their BP [12,13]. One billion people worldwide are believed to struggle with uncontrolled HTN [14].

In this study, our objective was to evaluate the knowledge of the Saudi population about the association between uncontrolled BP and HF. We noticed that patients with HTN did not have better knowledge than those without HTN. On the other hand, patients with HF had better knowledge. The main sources of information for the studied population were television and the internet. Interestingly, half of the participants did not recognize this fact. Many factors such as gender, occupation, knowledge about uncontrolled HTN, HF, and knowing what HF means affected the participant’s knowledge of the association between the two conditions. Unaware participants were less aware of certain symptoms related to HTN and HF than those who were aware. These symptoms include blurred vision, chest pain, headache, dizziness, and dyspnoea. Unaware participants also had a poorer understanding of relationship and lifestyle measures, with a better understanding of uncontrolled HTN leading to HF. Lifestyle measures for HTN control, such as reducing anxiety, medication compliance, increasing physical activity, and a low-sodium diet, were generally better understood by the informed group. Aware participants had different perceptions of risk factors, with diabetes mellitus, family history of HF, smoking, obesity, and HTN being the most common. Those without knowledge had lower percentages of each factor.

We found that 56.94% did not know about uncontrolled HTN, and nearly half were hypertensive. This finding should attract the attention of policymakers and stakeholders on the importance of providing health information to this population to raise their knowledge and control their disease condition. A similar study conducted in the USA assessed hypertensive patients' knowledge, awareness, and attitudes regarding HTN [10]. Findings showed that 90% of patients knew that lowering their BP would improve their health. However, 41% of the patients did not know their BP levels. Only 30% of the patients who correctly identified both SBP and DBP measures could correctly identify them; 27% of patients with elevated SBP and DBP perceived their BP as high. Likewise, Wolde et al. [15] assessed HTN knowledge among hypertensive patients in public health facilities in Gondar City. About 55.3% had low knowledge, 17.9% had moderate knowledge, and 26.8% had high knowledge. In the context of this study, age did not exhibit a significant association with possessing this information, whereas it was found that being male was significantly correlated. Interestingly, the existing literature has suggested that older age is often associated with a higher level of knowledge, unlike what was observed here [16,17]. However, we propose that using the internet and other sources of health information among the younger population could narrow this knowledge gap.

In this study, 50% of the participants knew that HTN was associated with the development of HF. The participant's understanding of the relationship between the two conditions was influenced by a variety of factors, including gender, occupation, awareness of uncontrolled HTN, HF, and understanding of what is meant by HF. Similar findings were reported by Lugo-Mata et al. [16]. They found different factors associated with knowledge about HTN, such as age, previous diagnosis of HTN, and family history. However, no association was found with gender, educational level, or body mass index. An important finding that should be considered is the channel selection that can be used to deliver health messages. We found that the internet and television were the most used media channels the studied population used to get health messages. These communication channels have proven very effective [17,18].

In the current study, we found that participants who were unaware of the HTN-HF link had less awareness of symptoms of HF, such as blurred vision, chest pain, headache, and dizziness. They also had a poorer understanding of relationship and lifestyle measures. The aware group better understood the lifestyle measures for HTN control, such as reducing anxiety, medication compliance, increasing physical activity, and a low-sodium diet. Similar findings were observed, aligning with what has been documented in the existing literature [16,19].

Strengths and limitations

The study emphasized a significant knowledge gap in the linkage between HTN and HF. However, the study has many limitations. A sampling bias through snowball sampling might lead to similarities among participants recruited through social networks. The study's focus on a specific region, Aseer, could limit the generalizability of findings to broader populations. Relying on self-reported data might introduce recall and reporting bias, especially regarding sensitive health information. Conducting the survey online might exclude those without internet access or technological familiarity, potentially skewing the sample. The online survey nature could attract health-conscious individuals, potentially introducing response bias. The cross-sectional design hampers the establishment of causality, revealing associations only at a one-time point.

## Conclusions

Patients with HTN did not show better awareness than those without HTN, while patients with HF demonstrated greater knowledge. The primary sources of health information were television and the internet. Gender, occupation, familiarity with uncontrolled HTN and HF, and understanding of the meaning of HF were all factors influencing participants' awareness of the relationship between the two conditions. Those unaware of the condition showed a lower recognition of specific symptoms linked to HTN and HF, including blurred vision, chest pain, headache, dizziness, and dyspnoea. Furthermore, their understanding of relationship and lifestyle interventions was poorer, with a weaker grasp of uncontrolled HTN leading to HF and the role of BP control in preventing HF. Understanding lifestyle measures for HTN management, such as reducing anxiety, adhering to medication, increasing physical activity, and adopting a low-sodium diet, was generally more pronounced in the aware group. The aware participants demonstrated distinct perceptions of risk factors, with diabetes mellitus, family history of HF, smoking, obesity, and HTN being the most commonly recognized factors.
